# Supplementary material for: Resolving the full spectrum of human genome variation using Linked-Reads
Source: Genome Res. 2019 Apr;29(4):635–45. doi: 10.1101/gr.234443.118 (PMC6442396; doi:10.1101/gr.234443.118)
Supplement: Supplemental Material [file supp_gr.234443.118_Supplemental_longranger-2.2.zip › longranger-2.2.tar.bz2!/lr2.2-public/lib/stan/cmdstan/stan/doc/api/html/classstan_1_1mcmc_1_1stepsize__var__adapter.html]

Stan: stan::mcmc::stepsize\_var\_adapter Class Reference


|  |  |
| --- | --- |
|  | Stan  2.5.0  probability, sampling & optimization |


- Main Page
- Related Pages
- Namespaces
- Classes
- Files

- Class List
- Class Index
- Class Hierarchy
- Class Members

All Classes Namespaces Files Functions Variables Typedefs Enumerations Enumerator Friends Macros Pages

- stan
- mcmc
- stepsize\_var\_adapter

Public Member Functions |
Protected Attributes |
List of all members

stan::mcmc::stepsize\_var\_adapter Class Reference

`#include <stepsize_var_adapter.hpp>`

Inheritance diagram for stan::mcmc::stepsize\_var\_adapter:

|  |  |
| --- | --- |
| Public Member Functions | |
|  | stepsize\_var\_adapter (int n) |
|  | |
| stepsize\_adaptation & | get\_stepsize\_adaptation () |
|  | |
| var\_adaptation & | get\_var\_adaptation () |
|  | |
| void | set\_window\_params (unsigned int num\_warmup, unsigned int init\_buffer, unsigned int term\_buffer, unsigned int base\_window, std::ostream \*e=0) |
|  | |
| Public Member Functions inherited from stan::mcmc::base\_adapter | |
|  | base\_adapter () |
|  | |
| virtual void | engage\_adaptation () |
|  | |
| virtual void | disengage\_adaptation () |
|  | |
| bool | adapting () |
|  | |

|  |  |
| --- | --- |
| Protected Attributes | |
| stepsize\_adaptation | stepsize\_adaptation\_ |
|  | |
| var\_adaptation | var\_adaptation\_ |
|  | |
| Protected Attributes inherited from stan::mcmc::base\_adapter | |
| bool | adapt\_flag\_ |
|  | |

## Detailed Description

Definition at line 12 of file stepsize\_var\_adapter.hpp.

## Constructor & Destructor Documentation

|  |  |  |  |  |  |
| --- | --- | --- | --- | --- | --- |
| stan::mcmc::stepsize\_var\_adapter::stepsize\_var\_adapter | ( | int | *n* | ) |  |

inline

Definition at line 16 of file stepsize\_var\_adapter.hpp.

## Member Function Documentation

|  |  |  |  |  |
| --- | --- | --- | --- | --- |
| stepsize\_adaptation& stan::mcmc::stepsize\_var\_adapter::get\_stepsize\_adaptation | ( |  | ) |  |

inline

Definition at line 19 of file stepsize\_var\_adapter.hpp.

|  |  |  |  |  |
| --- | --- | --- | --- | --- |
| var\_adaptation& stan::mcmc::stepsize\_var\_adapter::get\_var\_adaptation | ( |  | ) |  |

inline

Definition at line 23 of file stepsize\_var\_adapter.hpp.

|  |  |  |  |
| --- | --- | --- | --- |
| void stan::mcmc::stepsize\_var\_adapter::set\_window\_params | ( | unsigned int | *num\_warmup*, |
|  |  | unsigned int | *init\_buffer*, |
|  |  | unsigned int | *term\_buffer*, |
|  |  | unsigned int | *base\_window*, |
|  |  | std::ostream \* | *e* = `0` |
|  | ) |  |  |

inline

Definition at line 27 of file stepsize\_var\_adapter.hpp.

## Member Data Documentation

stepsize\_adaptation stan::mcmc::stepsize\_var\_adapter::stepsize\_adaptation\_

protected

Definition at line 42 of file stepsize\_var\_adapter.hpp.

var\_adaptation stan::mcmc::stepsize\_var\_adapter::var\_adaptation\_

protected

Definition at line 43 of file stepsize\_var\_adapter.hpp.

---

The documentation for this class was generated from the following file:

- src/stan/mcmc/stepsize\_var\_adapter.hpp


---

[ Stan Home Page ]

*© 2011–2014,
Stan Development Team.*
